# Efficient Catalytic Degradation of Phenol with Phthalocyanine-Immobilized Reduced Graphene–Bacterial Cellulose Nanocomposite

**DOI:** 10.3390/nano11092218

**Published:** 2021-08-28

**Authors:** Binbin Wu, Yikai Sun, Qiujin Fan, Jiahui Chen, Weizheng Fang, Shiliang Chen

**Affiliations:** Institute of Environmental Sciences, Qianjiang College, Hangzhou Normal University, Hangzhou 310018, China; wubb00718@sina.com (B.W.); sunyk010117@sina.com (Y.S.); fanqj000214@sina.com (Q.F.); chenjh010808@sina.com (J.C.); fangwz001226@sina.com (W.F.)

**Keywords:** bacterial cellulose, graphene, phthalocyanine, phenol, degradation

## Abstract

In this report, phthalocyanine (Pc)/reduced graphene (rG)/bacterial cellulose (BC) ternary nanocomposite, Pc-rGBC, was developed through the immobilization of Pc onto a reduced graphene–bacterial cellulose (rGBC) nanohybrid after the reduction of biosynthesized graphene oxide-bacterial cellulose (GOBC) with N_2_H_4_. Field emission scanning electron microscopy (FESEM) and Fourier transform infrared spectroscopy (FT-IR) were employed to monitor all of the functionalization processes. The Pc-rGBC nanocomposite was applied for the treatment of phenol wastewater. Thanks to the synergistic effect of BC and rG, Pc-rGBC had good adsorption capacity to phenol molecules, and the equilibrium adsorption data fitted well with the Freundlich model. When H_2_O_2_ was presented as an oxidant, phenol could rapidly be catalytically decomposed by the Pc-rGBC nanocomposite; the phenol degradation ratio was more than 90% within 90 min of catalytic oxidation, and the recycling experiment showed that the Pc-rGBC nanocomposite had excellent recycling performance in the consecutive treatment of phenol wastewater. The HPLC result showed that several organic acids, such as oxalic acid, maleic acid, fumaric acid, glutaric acid, and adipic acid, were formed during the reaction. The chemical oxygen demand (COD) result indicated that the formed organic acids could be further mineralized to CO_2_ and H_2_O, and the mineralization ratio was more than 80% when the catalytic reaction time was prolonged to 4 h. This work is of vital importance, in terms of both academic research and industrial practice, to the design of Pc-based functional materials and their application in environmental purification.

## 1. Introduction

Phenol and phenol derivatives are widely used in a series of fields such as petrochemicals, pharmaceuticals, textile manufacturing, wood products, steel foundries, etc. The resulting phenolic compound-containing effluents are classified as one of the highest priority types of industrial pollutants due to their toxicity to aquatic organisms and human beings, even at low concentrations [1,2]. The increasing concern regarding their environmental and health risks prompted the implementation of effective treatment techniques that are capable of dealing with phenolic compounds. Due to the toxicity and poor biodegradability of this kind of pollutant, biological degradation, which is prevalent in industrial wastewater treatment, is difficult to apply [3,4]. Other techniques, such as membrane filtration [5], solvent extraction [6,7] and chemical oxidation [8,9], have the drawbacks of low efficiency and the possible generation of toxic byproducts. Out of all the methods, adsorption is still considered as the most versatile, simple, and widely used treatment approach due to the low cost, high efficiency, and convenient operation of this method [10,11,12]. Among all of the adsorbents available for the adsorption of phenolic compounds, graphene stands out as an impressive candidate due to its unique physicochemical properties, including ultra-high surface area, environmental compatibility, good recyclability, and excellent mechanical strength [13,14,15]. There are studies on the adsorption of phenolic compounds onto graphene in the literature [13,16,17], and good adsorption capacity was reported. However, the adsorption method only transferred the phenolic compounds from the effluent to the adsorbent; for complete elimination of the pollutant, post-processing treatment was necessary before discharge into the environment.

The advanced oxidation process (AOP) is a research hotspot within the field of wastewater treatment because of the great potential of this technique in terms of the deep oxidation of organic compounds, including phenolic compounds, based on the generation of powerful oxidant hydroxyl radicals (·OH) [18,19,20,21,22]. The typical AOP, such as Fenton’s reagent (Fe^2+^ and H_2_O_2_), UV+H_2_O_2_, and UV+O_3_, has the disadvantages of having harsh application conditions, causing secondary pollution, and being unrecyclable. To solve these problems, different kinds of Fenton-like systems were developed by researchers [23,24,25,26], while highly efficient and industrial feasible reaction systems still need to be explored.

Thanks to their structural similarity with the naturally occurring metal porphyrin, metal phthalocyanine (Pc) complexes, a class of versatile functional molecules, were employed as bio-inspired catalysts, and the application of Pc as a Fenton-like catalyst was also frequently reported [27,28,29]. In practical applications, Pc was often prepared in the heterogeneous form for the prevention of the formation of inactive aggregates and the easy recovery of reaction media for recycling purposes [28,30,31,32,33]. Since the heterogeneous catalysis reaction often involves the diffusion of the reactants from the bulk phase to the external surface of the catalyst, adsorption of the reactants onto the catalyst, and catalytic reaction at specific active sites, the catalytic efficiency of the heterogeneous catalyst relies extensively on the adsorption process [34]. The improvement of the adsorption capacity of the heterogeneous catalyst can facilitate the subsequent catalytic reaction, and accordingly, the enhancement of the catalytic efficiency is reasonably expected.

By taking advantage of the excellent adsorption capacity of graphene and the high catalytic activity of Pc, ideal treatment of phenol can be achieved by simultaneous adsorption and catalytic degradation. The combinations of Pc together with graphene for the treatment of organic compounds were previously reported by us and others [35,36,37]. In this paper, a novel ternary nanocomposite, Pc-rGBC, was fabricated through the immobilization of Pc onto a reduced graphene–bacterial cellulose (rGBC) nanohybrid. The Pc-rGBC was applied for the treatment of phenol wastewater, and both the adsorption and catalytic degradation processes were thoroughly investigated. High performance liquid chromatography (HPLC), chemical oxygen demand (COD), and electron paramagnetic resonance (EPR) technologies were employed to study the catalytic oxidation mechanism of the Pc-rGBC+H_2_O_2_ reaction system.

## 2. Materials and Methods

### 2.1. Materials and Reagents

*Acetobacter xylinum* (category No. BNCC 280126) was purchased from BeNa Culture Collection Co., Ltd. (Beijing, China). Aqueous graphene oxide (GO) dispersion (0.5 mg/mL) was purchased from Aladdin Co., Ltd. (Shanghai, China). Sulfonated cobalt phthalocyanine (Pc, 98 wt%) was purchased from Energy Chemical Co., Ltd. (Shanghai, China). 5,5-dimethyl-1-pyrroline-N-oxide (DMPO), of analytical grade, was purchased from Sigma-Aldrich (Saint Louis, MO, USA). N, N-diethyl-p-phenylenediamine (DPD), D-glucose, ethanol, sodium hydroxide, dimethylformamide and other common chemical reagents were of analytical grade, and were purchased from Sinopharm Chemical Reagent Co., Ltd. (Beijing, China). Hydrogen peroxide (30 wt%) was obtained from Beijing Chemicals Co., Ltd. (Beijing, China). Acetonitrile, phosphoric acid, aromatic compounds, and organic acids, used for HPLC analysis, were of HPLC grade and were purchased from Sinopharm Chemical Reagent Co., Ltd. (Beijing, China).

### 2.2. Preparation of Pc-rGBC Nanocomposite

The bacterial strain *Acetobacter xylinum* was employed to produce both bacterial cellulose (BC) and graphene oxide-bacterial cellulose (GOBC). The culture medium for pristine BC was composed of 8.0% (w/v) D-glucose, 1.0% (w/v) yeast extract, 1.0% (w/v) ethanol, and disodium phosphate (Na_2_HPO_4_). For the preparation of GOBC nanohybrid, a GO-dispersed culture medium was first prepared. The GO concentration in culture medium was adjusted in the range of 0–200 mg/L. Typically, 125 mg/L of aqueous GO dispersion was mixed with 40 mL of BC culture medium, stirred for 120 min and sterilized at 121 °C in an autoclave for 30 min. The sterilized mixture was cultivated with the bacteria Acetobacter xylinum at 30 °C. The harvested BC and GOBC were incubated in a 0.10 mol/L NaOH solution for 30 min, and washed with ultrafiltration water until reaching a neutral pH. For the immobilization of Pc onto the GOBC nanohybrid, the sample was first immersed in a N_2_H_4_ solution (1 µL/mL) at 25 °C for 24 h, thoroughly washed with ultrafiltration water, and then the reduced graphene–bacterial cellulose nanohybrid (rGBC) was immersed into a saturated Pc solution and ultrasonicated for 24 h. The resulting product was washed several times with dimethylformamide (DMF) to eliminate the residual Pc, and then rinsed with ultrafiltration water. The GO content of GOBC and the Pc content of Pc-rGBC nanocomposite were calculated according to Equations (1) and (2), respectively:

(1) $$\mathrm{GO}\;\mathrm{content}\;\left(\mathrm{mg}/g\right)=\frac{m_{\mathrm{GO}}}{m_{\mathrm{GOBC}}}$$

(2) $$\mathrm{Pc}\;\mathrm{content}\;\left(µ\mathrm{mol}/g\right)=\frac{n_{\mathrm{Pc}}}{m_{\mathrm{Pc}-\mathrm{rGBC}}}$$

where *m*_GO_, *m*_GOBC_, and *m*_Pc−rGBC_ are the weights of GO (mg), GOBC membrane (g), and Pc-rGBC nanocomposite (g), respectively; *n*_Pc_ is the mole number of Pc in the nanocomposite (µmol), which was measured by atomic absorption spectrometry (Thermo Sollar M6).

### 2.3. Characterization

The morphologies of all samples were monitored by field emission scanning electron microscopy (FESEM, Serion, FEI, Hillsboro, Oregon, USA). Fourier transform infrared (FT-IR) spectra were carried out using a FT-IR spectrometer (Bruker Optics, Faellanden, Switzerland) with the KBr disc technique.

### 2.4. Adsorption and Catalytic Degradation of Phenol

The adsorption experiments were conducted in a stirred tank glass reactor and placed in a thermostatic water bath at 50 °C. Aqueous solution of phenol was used to simulate the wastewater. Phenol stock solution (100 mg/L) was prepared by dissolving phenol in deionized water and further diluted to the required concentrations before use. Typically, 1 mg of Pc-rGBC nanocomposite and 25 mL of phenol solution were mixed in a flask. After adsorption for a given time, samples were separated by filtration and the phenol concentrations were analyzed by UV-Vis spectroscopy at 270 nm. The concentration change of phenol solution was expressed as a change in *C*_t_*/C*_0_ value; the uptake of the adsorbate at time *t* (*q_t_* (mg/g)) and at equilibrium (*q_e_* (mg/g)) was calculated by Equations (3) and (4), respectively:

(3) $$q_{t}\;=\frac{C_{0}-C_{t}}{m_{\mathrm{Pc}-\mathrm{rGBC}}}\;\times V$$

(4) $$q_{e}\;=\;\frac{C_{0}-C_{e}}{m_{\mathrm{Pc}-\mathrm{rGBC}}}\;\times \;V$$

where *C*_0_, *C*_t_, and *C*_e_ are the concentrations (mg/L) of phenol at the initial time point, at time t, and at the point of equilibrium, respectively; *V* is the volume (L) of the solution; *m*_Pc−rGBC_ is the weight (g) of Pc-rGBC nanocomposite.

To initiate the catalytic oxidation process, a given volume of H_2_O_2_ was added into the above-mentioned mixture to make its initial concentration 50 mM. The concentration of H_2_O_2_ during the reaction was determined by a DPD photometric method [38]. The EPR signal of radicals formed during the reaction was spin-trapped by DMPO and examined with a Bruker-A300 X-band EPR spectrometer (Bruker, Karlsruhe, Germany). Analysis of the catalytic oxidation product was conducted by means of the HPLC method using an Agilent 1100 Series liquid chromatography device (USA) equipped with a UV/DAD detector. The HPLC series was equipped with a reverse-phase C18 5 µm column (150 mm in length, 4.6 mm in diameter), and the mobile phase comprised a mixture of acetonitrile and 2‰ phosphoric acid solution (35:965, *v*/*v*). The detection wavelength for the products was set at 214 nm. Identification was achieved through the comparison of retention times. The COD of the solution was measured using a 5B-3B (V8)-type COD analyzer (Lianhua, Beijing, China). The COD change of the solution was expressed as the change of the *COD*/*COD*_0_ value, where *COD* and *COD*_0_ are the instant COD and the initial COD value of the solution, respectively. The samples were filtrated with a 0.22 µm Millipore filter to remove any particles prior to examination by HPLC and with the COD analyzer. To test the stability of Pc-rGBC for cyclic runs, the nanocomposite was recycled after treatment of phenol wastewater, thoroughly washed with ultrafiltration water, and vacuum dried at 25 °C for 24 h for the next use. 

## 3. Results and Discussion

### 3.1. Materials’ Characterization

**Figure 1:**
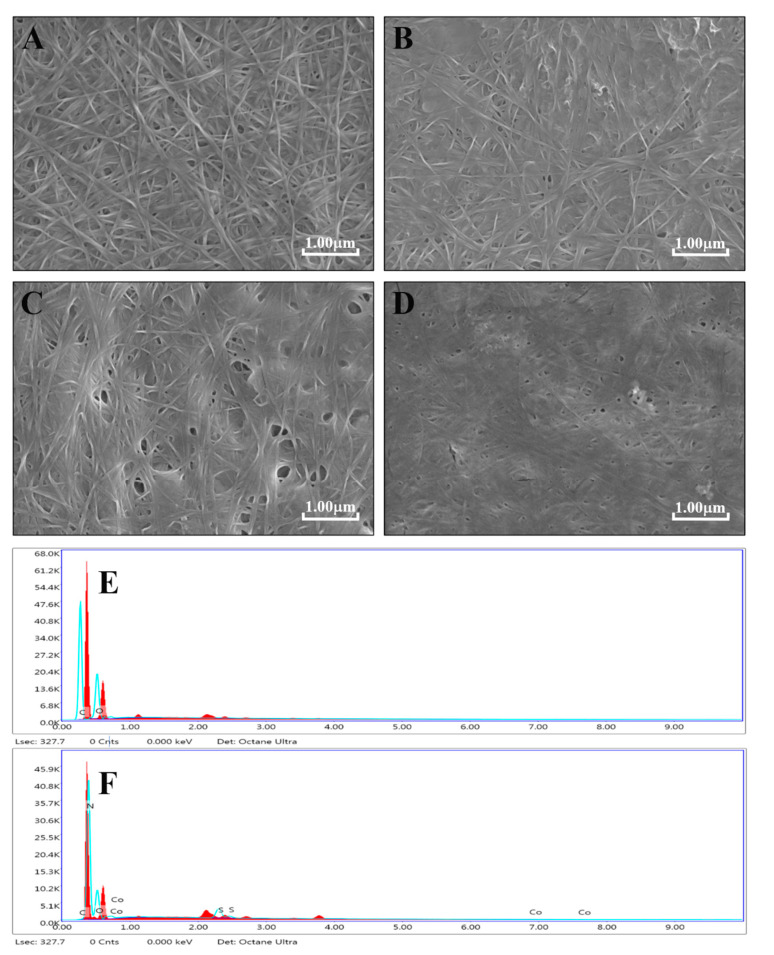
FESEM of (**A**): BC; (**B**): GOBC; (**C)**: rGBC; and (**D**): Pc-rGBC. EDX spectra of (**E**): rGBC; (**F**): Pc-rGBC.

Pc-rGBC nanocomposite was fabricated in three steps: the biosynthesis of GOBC, the reduction of GOBC to form rGBC, and the immobilization of Pc onto rGBC. The morphologies of BC, GOBC, rGBC, and Pc-rGBC were characterized by FESEM. For pure BC membrane, the diameter of nanofibers was several dozen nanometers, and a porous 3D structure was formed by these nanofibers (Figure 1A). The morphology of the GOBC membrane also has a nanoscale pattern, and the obvious adsorption of GO onto BC nanofibers was observed (Figure 1B). After the reduction of GOBC with N_2_H_4_, little change of morphology of rGBC was found, implying that there was good membrane stability during the chemical treatment (Figure 1C). When Pc was immobilized onto rGBC, the resulting Pc-rGBC membrane showed a much coarser morphology (Figure 1D). Furthermore, compared with that of rGBC, the existence of the Co atom and the S atom for the Pc-rGBC nanocomposite was confirmed by EDX spectra (Figure 1E,F), which directly indicated the successful immobilization of Pc onto the rGBC membrane.

**Figure 2:**
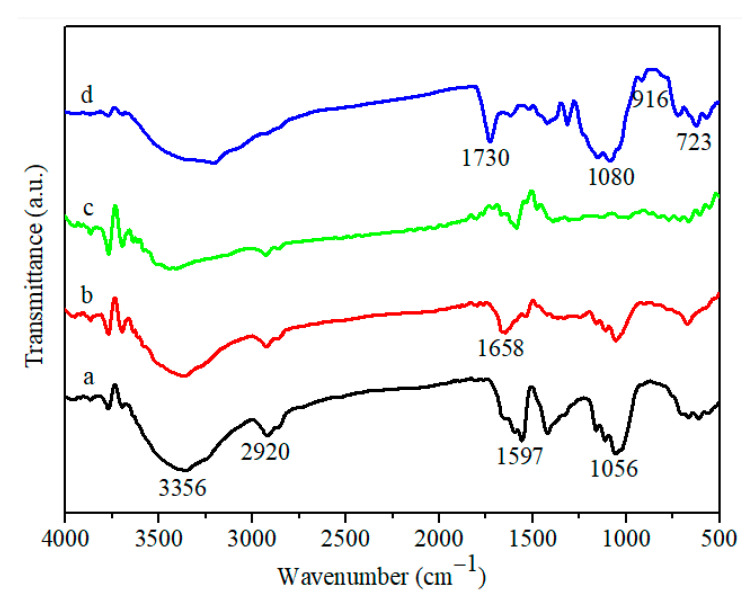
FT-IR spectra of (a): BC; (b): GOBC; (c): rGBC; and (d): Pc-rGBC.

Fourier transform infrared (FT-IR) absorption spectra were employed to identify the vibration of the functional groups of all of the prepared membranes. For the BC membrane, the strong absorption located at around 3356 cm^−1^ was the typical -OH stretching vibration (Figure 2a). The presence of abundant hydrophilic oxygen-containing groups of BC was also verified by the peaks at 1597 cm^−1^ (–OH bending vibration) and 1056 cm^−1^ (C–O–C vibration), and the peak at 2920 cm^−1^ was attributed to the C–H vibration. For GOBC, the new peak located at 1658 cm^−1^ could be ascribed to the C=O vibration, which was the result of the successful incorporation of GO in the BC membrane (Figure 2b) [39]. After the reduction treatment, the peak ascribed to the C=O functional groups suffered a significant decrease in intensity, revealing the successful removal of C=O with the reduction of N_2_H_4_ (Figure 2c). For Pc-rGBC nanocomposite, the characteristic peaks located at 916 cm^−1^ and 723 cm^−1^ were related to metal ion and Pc ring, which denotes the coordination between the three-dimensional unoccupied orbital of the Co atom and the N atoms in the Pc rings [40]. Furthermore, the characteristic peaks at 1730 cm^−1^ and 1080 cm^−1^ were attributed to the C=N stretching vibration and the S=O stretching vibration of Pc, respectively (Figure 2d). All these results further verified the successful preparation of Pc-rGBC nanocomposite.

### 3.2. Study of Cultivation and Pc Immobilization Process

**Figure 3:**
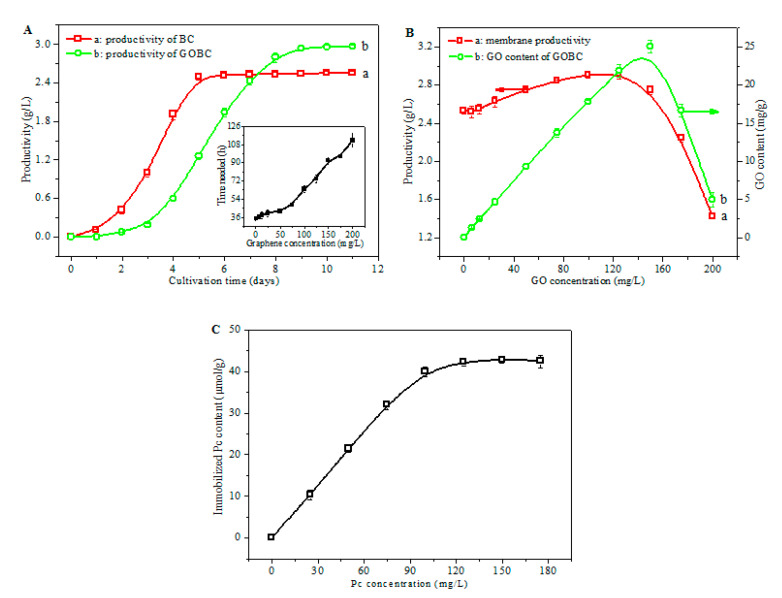
(**A**): Influence of cultivation time on the productivity of (a): BC; (b): GOBC membrane; the inset showed the influence of intial GO concentration on the time needed to produce 0.3 g/L of membrane. (**B**): Influence of initial GO concentration on (a): the productivity of the membrane; (b): the GO content of the membrane (cultivation time was set as 10 d). (**C**): Influence of Pc concentration on the immobilized Pc content of Pc-rGBC nanocomposite (the GO content of the GOBC membrane was set as 2.92 g/L).

To adequately understand the membrane production process, the time course of the productivity of the BC and the GOBC membrane during cultivation was studied (Figure 3A). The production of the BC membrane (Figure 3A (a)) started slowly, and small nanofibers were found in the culture medium in the first 2 days; this period was known as the lag phase of the bacteria [41]. The bacteria reached the exponential growth phase within 2 days of culture; accordingly, the productivity of BC increased rapidly. After cultivation for 5 days, ca. 2.5 g/L of BC membrane was produced; a further increase in cultivation time has little effect on the productivity of the membrane, which was mainly due to the complete consumption of the substrate. In comparison, the production of the GOBC membrane was initiated more slowly than that of pure BC (Figure 3A (b)). After incubation, the bacteria needed to adapt to the new environmental conditions and accommodate for a fast division. Due to the presence of GO nanosheets in the culture medium, the growth of the bacteria was affected to some extent [42,43]; therefore, a prolonged lag phase was observed. With 125 mg/L of GO added into the culture medium, less than 0.2 g/L of GOBC membrane could be produced for cultivation for 3 days, implying that the cultivation was inhibited to some extent by GO. It was reasonable that this inhibition effect had an increasing trend with the increase in GO concentration (inset of Figure 3A). It took ~75 h to produce 0.3 g/L (10% of the maximum productivity) of membrane in the culture medium with 125 mg/L of GO, compared to only ~35 h for the membrane without graphene. Further increases in graphene concentration resulted in much more severe inhibitions of membrane growth; the cultivation process was drastically inhibited when the GO concentration was higher than 200 mg/L.

The influence of the initial GO concentration on the productivity and GO content of the membrane is shown in Figure 3B. Without the addition of GO, ca. 2.5 g/L of membrane can be formed within 5 days of cultivation (Figure 3B (a)). When the GO concentration was increased, a slight increase in membrane productivity was observed, which can be ascribed to the incorporation of GO in the membrane. The productivity of the membrane was 2.92 g/L when the GO concentration in the culture medium was 125 mg/L, which was 15% higher than that without the addition of GO. However, further increases in GO concentration resulted in significant decreases in membrane productivity. With the addition of 200 mg/L of GO in the culture medium, the productivity drastically decreased to 1.42 g/L, which was only ca. 50% of the maximum value. The production of GOBC needed to include the following processes: firstly, BC nanofibers were synthesized by the bacteria, and GO was then adsorbed onto the formed nanofibers; subsequently, the newly formed nanofibers were covered on the GO nanosheet, and, to a certain extent, the bacteria was separated from GOBC and another cultivation process was started [44]. By experiencing several circles of nanofiber synthesis–GO adsorption–nanofiber coverage–bacteria separation, GOBC membrane was produced. When the GO concentration in the culture medium was too high, the bacteria had a strong tendency to be trapped by GO nanosheets, and both the nanofiber synthesis and the bacteria separation step were severely hindered; consequently, the whole cultivation process was stopped.

A similar trend in terms of variation was found for the GO content of the membrane, which increased almost linearly in the GO concentration range between 0 and 125 mg/L (Figure 3B (b)), implying a nearly complete incorporation of GO in the membrane during the cultivation process. The GO content decreased drastically when the initial GO concentration was higher than 150 mg/L (Figure 3B (b)), which was in accordance with the above analysis, i.e., an excessive GO concentration results in the early termination of the cultivation process. Considering both the productivity and GO content of the membrane, the optimal condition for membrane production was as follows: a cultivation time of 10 d and an initial GO concentration of 125 mg/L.

The effect of the initial Pc concentration on the immobilized Pc content of the Pc-rGBC nanocomposite was also studied, and the result is shown in Figure 3C. The Pc content of the Pc-rGBC nanocomposite increased rapidly with the increasing of the initial Pc concentration. When 120 mg/L of Pc solution was used, the resulting Pc content of Pc-rGBC was ca. 40 µmol/g. This value stayed almost constant with further increases in the initial Pc concentration, implying that the graphene nanosheets were already saturated by Pc molecules.

### 3.3. Adsorption and Catalytic Oxidation of Phenol with Pc-rGBC

**Figure 4:**
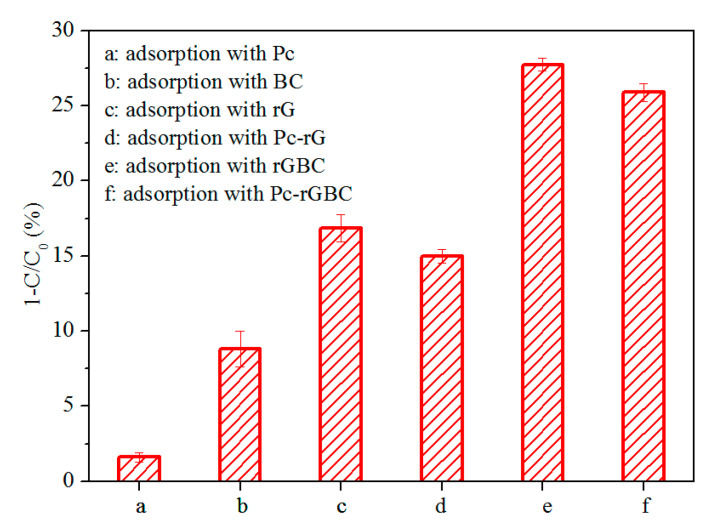
Comparison chart of phenol concentration change adsorbed by a: Pc; b: BC; c: rG; d: Pc-rG; e: rGBC; and f: Pc-rGBC. Initial phenol concentration = 100 mg/L; T = 50 °C; adsorption time = 180 min.

The prepared Pc-rGBC nanocomposite was intended to be a heterogeneous catalyst for the catalytic decomposition of organic compounds, with phenol chosen as a model target. It is well known that a heterogeneous catalytic reaction often begins with the diffusion and adsorption of the reactants to the active sites of the heterogeneous catalyst [34,45]; thus, the adsorption behavior of the nanocomposite should be thoroughly examined. The adsorption capacity of different combination parts of Pc-rGBC to the phenol solution was shown in Figure 4. A slight decrease in phenol concentration was found when Pc was added into the solution (Figure 4 (a)). With the addition of BC, ca. 10% of the phenol could be adsorbed (Figure 4 (b)), suggesting that the BC substrate was accessible to phenol, which was important for the subsequent heterogeneous catalytic reaction. A greater than 15% decrease in phenol solution was observed with rG employed as an adsorbent (Figure 4 (c)), implying that rG has good affinity to phenol molecules. A slightly lower adsorption capacity of Pc-rG was observed compared with that of rG (Figure 4 (d)). Interestingly, an obvious decrease in phenol concentration was achieved when rGBC was added as an adsorbent. Approximately 28% of the phenol was effectively removed after the adsorption process reached a dynamic equilibrium (Figure 4 (e)). The concentration of phenol also underwent a ca. 25% decrease with the addition of Pc-rGBC. The high adsorption capacity of Pc-rGBC was the result of the synergistic effect of BC and rG: besides its good accessibility to phenol molecules, BC substrate can also improve the dispersion of graphene, which, in return, promotes the adsorption of phenol; thus, the adsorption amount of phenol was greatly increased.

**Figure 5:**
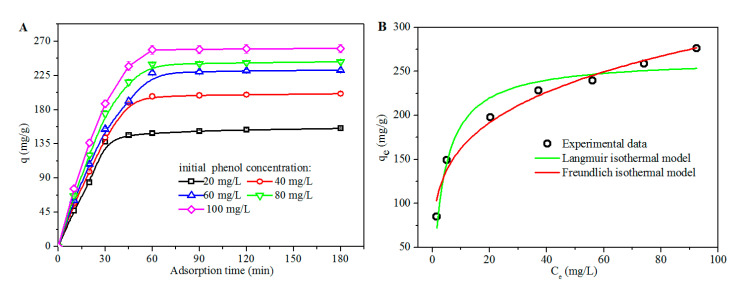
(**A**): The influence of adsorption time on the q values of Pc-rGBC at various initial phenol concentrations at 50 °C. (**B**): Equilibrium adsorption isotherm of phenol adsorbed onto Pc-rGBC at 50 °C. Points: experimental data; red line: Freundlich model; blue line: Langmuir model.

The effect of the adsorption time on the adsorbed amount of phenol per unit of weight of Pc-rGBC (q value), with respect to different initial concentrations of phenol, is shown in Figure 5A. For an initial phenol concentration of 20 mg/L, a rapid increase in q value was observed, revealing that phenol molecules were quickly adsorbed onto the Pc-rGBC nanocomposite. The adsorption reached a dynamic equilibrium within 45 min, and the q_e_ value was ca. 150 mg/g, i.e., 75% of phenol molecules were adsorbed onto the Pc-rGBC. With the increase in initial phenol concentration, the time required to reach dynamic adsorption equilibrium was slightly increased. For an initial phenol concentration of 100 mg/L, the q_e_ value was as high as 260 mg/g when the adsorption of Pc-rGBC was saturated after 60 min.

The adsorption isotherm models were employed to describe the interaction between the adsorbent (Pc-rGBC) and the adsorbate (phenol). The data for adsorption isotherms were obtained after the adsorption reached dynamic equilibrium at 50 °C (Figure 5B). The Langmuir and Freundlich isotherm models, which are suitable for the monolayer adsorption of the adsorbate onto a surface with a finite number of identical and equivalent sites, and onto a heterogeneous surface with multilayer adsorption, respectively, were adopted to analyze the adsorption behavior of Pc-rGBC. It seems that the Freundlich model fitted well with the phenol adsorption result, with the correlation coefficient (R^2^) reaching 0.99, which implyied that phenol was adsorbed onto Pc-rGBC in the form of multilayer adsorption, and thus, that multiple adsorption patterns may be responsible for this adsorption behavior.

**Figure 6:**
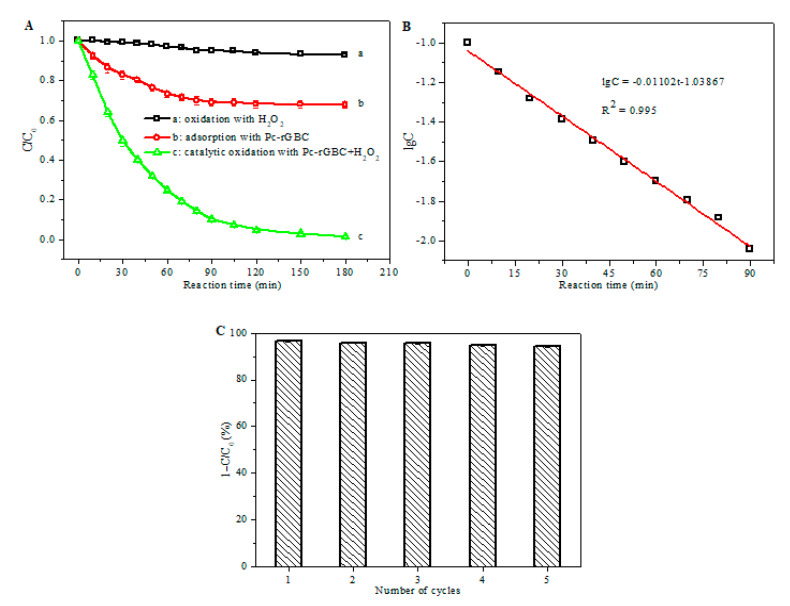
(**A**): Concentration changes of phenol (initial concentration 100 mg/L) under different reaction conditions: a H_2_O_2_ (50 mM); b Pc-rGBC (1 mg); c Pc-rGBC (1 mg) and H_2_O_2_ (50 mM). (**B**): lgC of H_2_O_2_ as a function of reaction time at 50 °C. (**C**): Repetitive catalytic oxidation of phenol (initial concentration 100 mg/L) with Pc-rGBC (1 mg) and H_2_O_2_ (50 mM); reaction time = 120 min.

The catalytic performance of the Pc-rGBC nanocomposite for the treatment of phenol wastewater was further studied, with H_2_O_2_ used as an oxidant. As shown in Figure 6A, no obvious change in the concentration of phenol was observed with H_2_O_2_ (Figure 6A (a)), implying that phenol molecules were difficult to oxidize with H_2_O_2_ alone. When Pc-rGBC was present, a gradual decrease in phenol concentration was found, which was ascribed to the good adsorption capacity of Pc-rGBC in relation to the phenol molecules. A dynamic equilibrium of the adsorption was reached within 90 min; ca. 30% of phenol could be effectively adsorbed onto the Pc-rGBC (Figure 6A (b)). In comparison, a rapid decrease in phenol concentration was observed when both Pc-rGBC and H_2_O_2_ were present (Figure 6A (c)). More than 90% of the phenol was efficiently removed within 90 min, and the value was as high as 99% when the reaction time was prolonged to 180 min. This result indicated that the Pc-rGBC+H_2_O_2_ was an excellent catalytic system for the elimination of phenol wastewater.

The relationship between the logarithm of concentration of H_2_O_2_ (lgC) and the reaction time in the presence of Pc-rGBC at 50 °C is shown in Figure 6B, and a good linear correlation was found with a correlation coefficient of R^2^ = 0.995. Similar results can also be found at reaction temperatures of 30 °C, 40 °C, 60 °C, and 70 °C (Figures S1–S4), revealing that the decomposition of H_2_O_2_ by the Pc-rGBC catalyst followed a pseudo-first order decay kinetics during the catalytic oxidation, which was in accordance with similar reaction systems [46,47,48].

The recycling performance of the heterogeneous catalyst is of vital importance to its practical application. To investigate the stability of the Pc-rGBC nanocomposite, the recycling experiment of its catalytic oxidation performance was evaluated, and the results are shown in Figure 6C. It is clear that little decline in the catalytic activity of Pc-rGBC was found; more than 94% of the phenol could be effectively decomposed within 120 min after repetitive recycling for five times. This result indicated that the Pc-rGBC nanocomposite has excellent recycling performance and is promising for the practical treatment of phenol-like organic wastewater.

**Figure 7:**
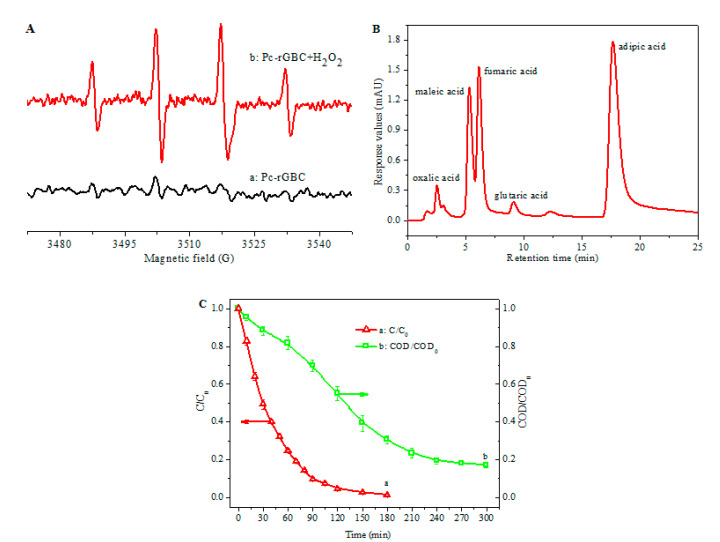
(**A**): Electron paramagnetic resonance (EPR) spectra of phenol solution in the presence of a: Pc-rGBC and b: Pc-rGBC+H_2_O_2_. (**B**): HPLC of the products of the catalytic oxidation of phenol with the catalytic reaction system. (**C**): Concentration and COD changes in the phenol solution in the catalytic oxidation of phenol by Pc-rGBC+H_2_O_2_.

The EPR technique was used to detect the active species formed during the reaction. As shown in Figure 7A (a), no EPR signal was found in the absence of H_2_O_2_. For comparison, four characteristic peaks of DMPO-·OH, with an intensity of 1:2:2:1, appeared with the presence of both Pc-rGBC and H_2_O_2_, revealing the formation of ·OH radicals (Figure 7A (b)). Thanks to the high adsorption capacity of Pc-rGBC and the formation of highly reactive ·OH, phenol can thus be rapidly decomposed with the Pc-rGBC+H_2_O_2_ reaction system. 

In order to further investigate the catalytic oxidation mechanism of phenol with Pc-rGBC+H_2_O_2_, the products formed in the catalytic reaction were examined by HPLC (Figure 7B). By comparing the peaks of the HPLC chromatogram with standard samples (see Figure S5), which were qualified by retention time, several major residual organic acids were identified: oxalic acid (RT = 2.533 min), maleic acid (RT = 5.207 min), fumaric acid (RT = 6.193 min), glutaric acid (RT = 9.146 min), and adipic acid (RT = 17.893 min). It is worthwhile to note that the retention times of succinic acid (Figure S5C) and muconic acid (Figure S5F) are very close to those of maleic acid (Figure S5B) and adipic acid (Figure S5G), and thus, succinic acid and muconic acid may also be considered as the catalytic oxidation intermediates of phenol; detailed analysis will be performed in the next work.

Figure 7C shows the changes in the phenol concentration and the COD value of the solution during the catalytic oxidation of phenol by the Pc-rGBC+H_2_O_2_ reaction system. As can be observed, ca. 90% of the phenol could be catalytic oxidized within only 90 min (Figure 7C (a)); accordingly, the COD removal efficiency was 30% (Figure 7C (b)),which was in accordance with the HPLC results, i.e., organic acids existed as intermediates of the catalytic oxidation of phenol. When the reaction time was prolonged to 240 min, the COD removal efficiency reached ca. 80%; in other words, more than 80% of the phenol was deeply mineralized to CO_2_ and H_2_O.

Based on the above experimental results, the following reaction mechanism was proposed for the catalytic decomposition of phenol by Pc-rGBC+H_2_O_2_: firstly, phenol was diffused from solution and adsorbed onto the Pc-rGBC nanocomposite; secondly, H_2_O_2_ was catalyzed by Pc-rGBC to form hydroxyl radicals; thirdly, the formed ·OH attacked the aromatic ring of the phenol, and the ring-opening reaction gave rise to the formation of organic acids such as oxalic acid, maleic acid, fumaric acid, glutaric acid, and adipic acid. As the catalytic oxidation proceeded, some of these organic compounds were further mineralized to CO_2_ and H_2_O, and the mineralization ratio was more than 80% for 4 h of catalytic oxidation. 

## 4. Conclusions

In summary, in the present study, the Pc catalyst was immobilized onto the reduced GOBC nanohybrid for the fabrication of Pc-rGBC nanocomposite. The Pc-rGBC showed a high adsorption capacity for the treatment of phenol wastewater. With H_2_O_2_ used as an oxidant, Pc-rGBC could catalytically decompose phenol molecules with high efficiency; the concentration decreased by more than 99% within 180 min of catalytic oxidation. Organic acids, such as oxalic acid, maleic acid, fumaric acid, glutaric acid, and adipic acid, were formed during the reaction; some of these intermediates could be further mineralized to CO_2_ and H_2_O, and the mineralization ratio reached 80% when the reaction time was prolonged to 4 h.

## Supplementary Materials

The following are available online at https://www.mdpi.com/article/10.3390/nano11092218/s1, Figures S1–S4: lgC of H_2_O_2_ as a function of reaction time at 30 °C, 40 °C, 60 °C and 70 °C, respectively. Figure S5: Standard HPLC chromatograms for A: oxalic acid, B: maleic acid, C: succinic acid, D: fumaric acid, E: glutaric acid, F: muconic acid, and G: adipic acid.
