# Supplementary material for: Efficient Catalytic Degradation of Phenol with Phthalocyanine-Immobilized Reduced Graphene–Bacterial Cellulose Nanocomposite
Source: Nanomaterials (Basel). 2021 Aug 28;11(9):2218. doi: 10.3390/nano11092218 (PMC8465619; doi:10.3390/nano11092218)
Supplement: Supplementary file 1 [file nanomaterials-11-02218-s001.zip › nanomaterials-1337758-supplementary.pdf]

nanomaterials

Supplementary Materials

Efficient Catalytic Degradation of Phenol with Phthalocyanine-Immobilized Reduced Graphene–Bacterial Cellulose Nanocomposite

Binbin Wu, Yikai Sun, Qiujin Fan, Jiahui Chen, Weizheng Fang and Shiliang Chen *

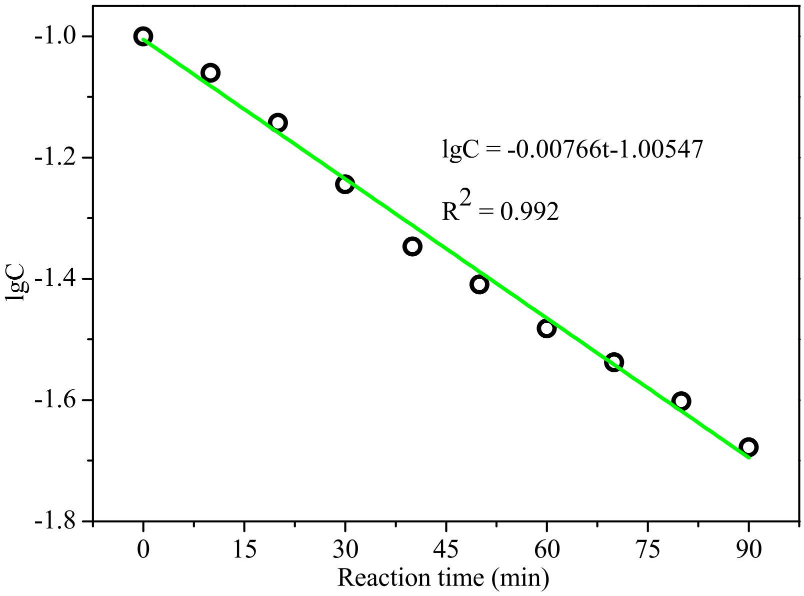

Figure S1. lgC of H_2O_2 as a function of reaction time at 30 °C.

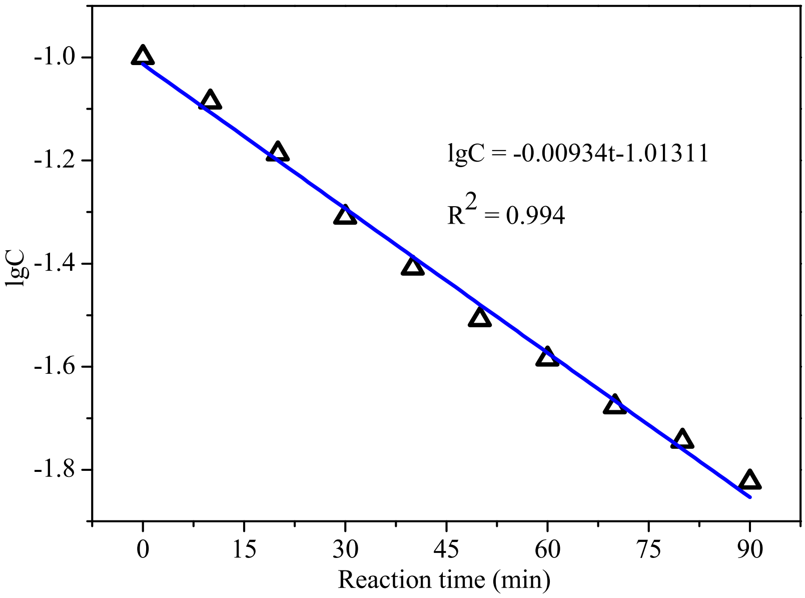

Figure S2. lgC of H_2O_2 as a function of reaction time at 40 °C.

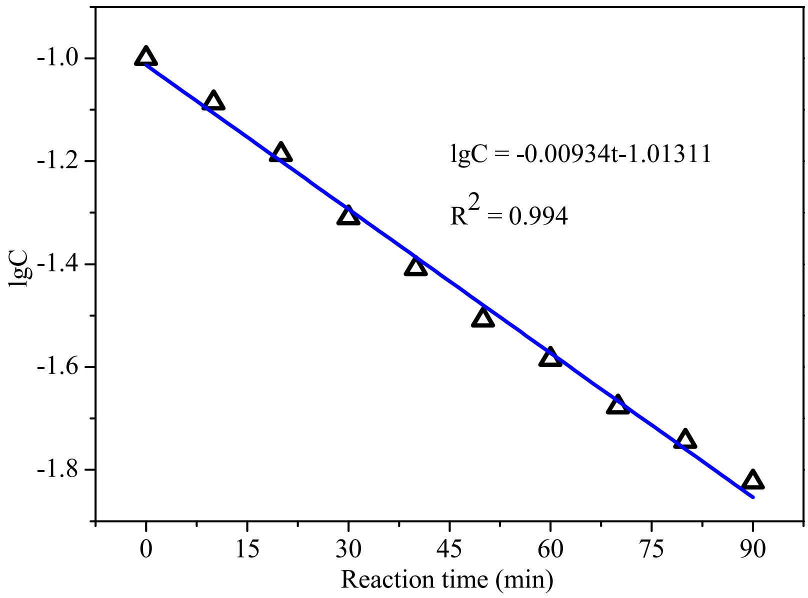

Figure S3. lgC of H_2O_2 as a function of reaction time at 60 °C.

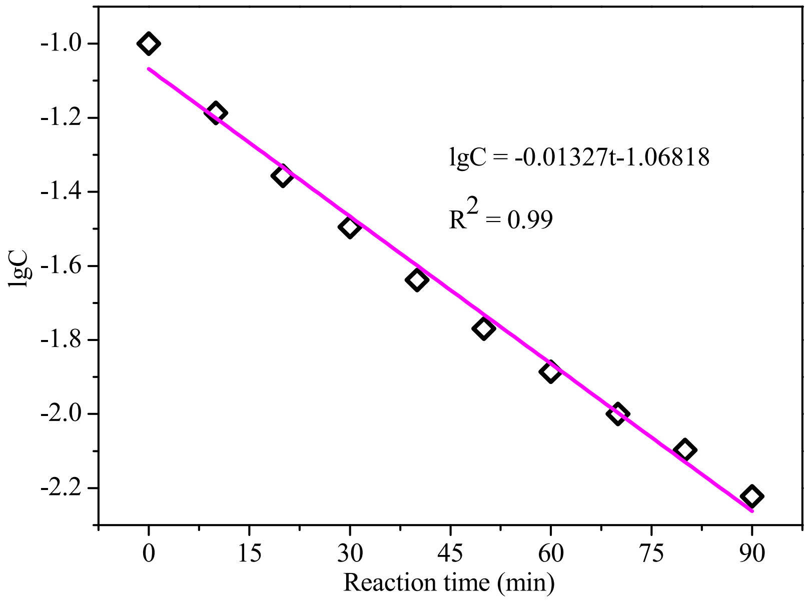

Figure S4. lgC of H_2O_2 as a function of reaction time at 70 °C.

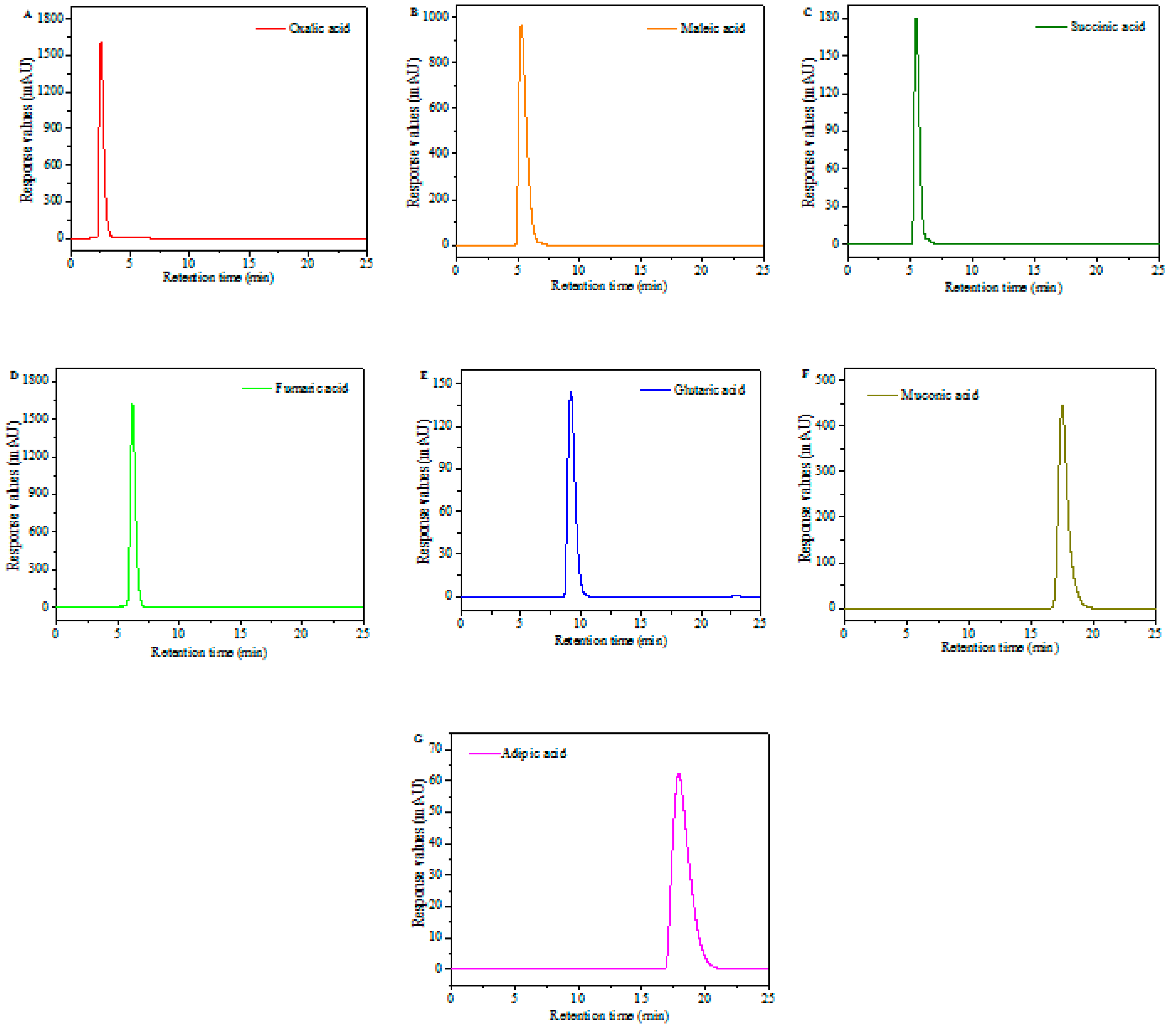

Figure S5. Standard HPLC chromatograms for A: oxalic acid, B: maleic acid, C: succinic acid, D: fumaric acid, E: glutaric acid, F: muconic acid, and G: adipic acid.
